# The different benefits and side effects of nivolumab combined with ipilimumab in diverse cancer: A Protocol for Systematic Review

**DOI:** 10.1097/MD.0000000000019367

**Published:** 2020-03-13

**Authors:** Hongxuan Tong, Yu Hao, Kaili Wang, Lekang Xiang, Tao Lu

**Affiliations:** a Chinese Medicine, Beijing, China School of Life Sciences, Beijing University of Chinese Medicine; b Institute of Basic Theory for Chinese Medicine, China Academy of Chinese Medical Sciences, Beijing, China; c School of Traditional Chinese Medicine, Beijing University of Chinese Medicine Beijing University of Chinese Medicine.

**Keywords:** cancer, ipilimumab, nivolumab, protocol, randomized controlled trials, systematic review

## Abstract

**Introduction:**

This systematic review protocol aims to provide the methods used to assess the total benefits and side effects in all cancer patients and their respective benefits and side effects in different cancers.

**Methods and analysis:**

The following electronic bibliographic databases will be selected without any language restriction: PubMed, EMBASE, The Cochrane Library, Scopus and Web of Science without an upper-limit date until July 12, 2019. Searches will also be performed in the following trials registers: ClinicalTrials.gov (www.ClinicalTrials.gov), the ISRCTN registry (www.isrctn.com), the WHO International Clinical Trials Registry Platform (www.who.int/trialsearch/Default.aspx) and the EU Clinical Trials Register (www.clinicaltrialsregister.eu). All randomized controlled trials related to the combination of nivolumab and ipilimumab for cancer patients will be included. Outcomes will include curative effect, chemotherapeutic response rate, adverse events. Study inclusion, data extraction and quality assessment will be performed independently by two reviewers. Assessment of risk of bias and data synthesis will be performed using Review Manager software.

**Ethics and dissemination:**

Ethics approval is not required because individual patients’ data are not included. The findings of this systematic review will be disseminated through peer-reviewed publication.

**PROSPERO registration number:**

CRD42018109732

## 1 Introduction

For the past several decades, immunotherapy has been 1 of the most important and promising therapies for cancer gradually. As 1 type of immunotherapy, immune checkpoint inhibitors have achieved unprecedented clinical success in many types of cancers and has led to a treatment revolution in the field of cancer immunotherapy. With this approach, specific monoclonal antibodies block suppressive ‘checkpoint’ receptors, thereby stimulating T cell function and thus enhancing antitumor immunity, such as cytotoxic T-lymphocyte antigen-4 (CTLA-4) and programmed death protein-1 (PD-1).^[1]^

CTLA-4, is an inhibitory molecule expressed on T cell. Rather than stimulating T cells, however, CTLA-4 provides inhibitory signals to the T cell, serving as a negative feedback loop.^[2–4]^ Theoretically, blocking CTLA-4 using McAbs (monoclonal antibody) may sustain the activation and proliferation of tumor-specific T cells, so it can initiate the development of an effective tumor-specific immune response. Ipilimumab, 1 type of monoclonal antibodies against human cytotoxic T-lymphocyte protein 4 (CTLA-4), showed an exciting therapeutic effect in melanoma, lung cancer, prostate cancer as a single dose or combination with other chemotherapy.^[5–7]^

The programmed death protein-1 (PD-1) pathway is also an immune checkpoint which can regulate differentiation and apoptosis of T cell.^[8]^ As the most important member, PD-1 is a protein receptor not only expressed in T cells, but also expressed in B cells, dendritic cells, natural killer T cells, and activated monocytes.^[9–11]^ As to its downregulation of immune response especially in cancer, PD-1 inhibitors which block the binding with PD-L1 and PD-L2 can interdict tumor-generated immunosuppress.^[8,12]^ Nivolumab, one type of monoclonal antibodies against human PD-1, also reveals its anti-tumor potential in melanoma, lung cancer, colorectal cancer, and advanced gastric or gastro-oesophageal junction cancer.^[13–15]^

Although ipilimumab and nivolumab have been proven their therapeutic effect in several clinical trials as new strategies, only part of cancer patients can benefit from these 2 agents. Meanwhile, drug tolerance remains a confusing problem and therapeutic challenge during the treatment.^[16]^ Thus, the combination of these 2 agents may be another approach to further improve the therapeutic effect. Based on recent clinical trials using the combination of these 2 agents in the treatment of multiple cancers, it is very necessary to assess the total benefits and side effects in all cancer patients and their respective benefits and side effects in different cancers.

### 1.1 Review question

(1) How much do the total and respective benefits obtain from the combination of ipilimumab and nivolumab in different types of cancers?
(2) How much do the total and respective side-effects obtain from the combination of ipilimumab and nivolumab in different types of cancers?

### 1.2 Objectives

Our systematic review aims to evaluate the **benefits** and **safety** of the **combination of ipilimumab and nivolumab** for cancer patients. For this purpose, following comparisons will be addressed:

(1) Determine the **total benefits** from **ipilimumab combined with nivolumab** versus single agent or other types of chemotherapy in all types of cancers.
(2) Determine the **respective benefits** from **ipilimumab combined with nivolumab** versus single agent or other types of chemotherapy in different cancers.
(3) Determine the **total side-effects** from **ipilimumab combined with nivolumab** versus single agent or other types of chemotherapy in all types of cancers.
(4) Determine **respective side-effects** from **ipilimumab combined with nivolumab** versus single agent or other types of chemotherapy in different cancers.

## 2 Methods and dedign

This systematic review and meta-analysis was conducted according to the Preferred Reporting Items for Systematic Reviews and Meta-Analyses Statement (PRISMA) guidelines^[17]^ and has been registered on the web of PROSPERO (https://www.crd.york.ac.uk/PROSPERO/display_record.php?ID=CRD42018109732). Our research team consisted of clinicians and methodologists, all of whom have contributed to the design of this study.

### 2.1 Criteria for considering studies for the review

#### 2.1.1 Types of participants

We will include all participants who suffered from any kind of cancer regardless of their country, age, sex, and ethnicity.

#### 2.1.2 Types of studies

We will only include RCTs in which nivolumab was combined with ipilimumab cancer patients without any language restrictions. Non-RCTs and uncontrolled clinical trials will be excluded.

#### 2.1.3 Types of interventions

Patients who were diagnosed as cancer were treated with the combination of nivolumab and ipilimumab. No concern the order of administration and the dosage change during treatment. Patients treated with nivolumab and Ipilimumab (experimental group) irrespective of that whether it is the first-line treatment. Meanwhile, patients who were also prescript with other similar drug or combined with other chemotherapeutic drugs will be excluded.

Control interventions, including the single use of nivolumab or ipilimumab or any other kind of chemotherapy. Trials evaluating the nivolumab or ipilimumab plus another similar drug (McAb) will be excluded

#### 2.1.4 Types of outcomes

We will consider included studies reporting the following outcomes with enough data to compute these estimates:

(1) Curative effect: Treatment outcomes in 6-month OS rate, 1-year OS rate, 6-month PFS rate, 1-year PFS rate;
(2) Chemotherapeutic response rate: complete response rate, partial response rate, overall response rate, disease control rate, progressive disease
(3) Adverse events (AEs): fatigue, diarrhea, nausea, vomiting, decreased appetite, headache, maculopapular rash, increased ALT level, increased AST level, increased lipase level, increased amylase level.

### 2.2 Search methods for identification of studies

#### 2.2.1 Electronics searches

The following electronic bibliographic databases will be selected without any language restriction: PubMed, EMBASE, The Cochrane Library, Scopus and Web of Science (Science and Social Science Citation Index) without an upper-limit date until July 12, 2019. Searches will also be performed in the following trials registers: ClinicalTrials.gov (www.ClinicalTrials.gov), the ISRCTN registry (www.isrctn.com), the WHO International Clinical Trials Registry Platform (www.who.int/trialsearch/Default.aspx) and the EU Clinical Trials Register (www.clinicaltrialsregister.eu).

We will also search the references of all relevant articles including systematic reviews and literature reviews to identify other eligible studies in case of the missing during our search, and then their full texts will be retrieved. Experts in the field will be contacted for additional published and unpublished studies. There is no upper-limit date until July 12, 2019, and there are also no language restrictions.

The search strategy will include only terms relating to or describing the intervention. The following terms will be searched: nivolumab, MDX-1106, ONO-4538, BMS-936558, Opdivo, Ipilimumab, Yervoy, MDX 010, MDX010, MDX-010, MDX-CTLA-4, MDX CTLA 4. The search strategy for PubMed is shown in Table 1.

**Table 1:**
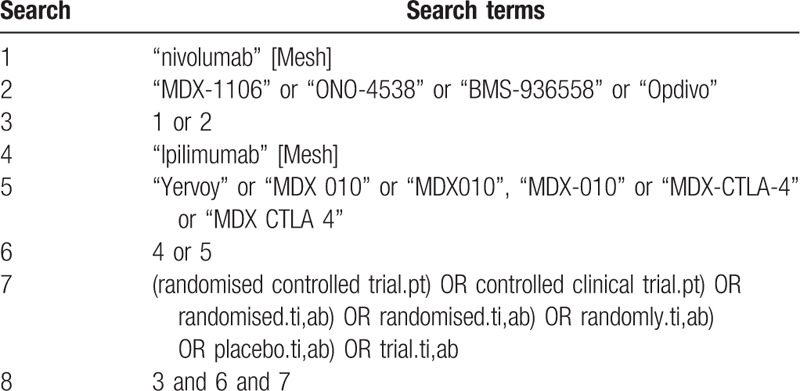
Search strategy used in PubMed database.

### 2.3 Data collection and analysis

#### 2.3.1 Selection of studies

**Figure 1:**
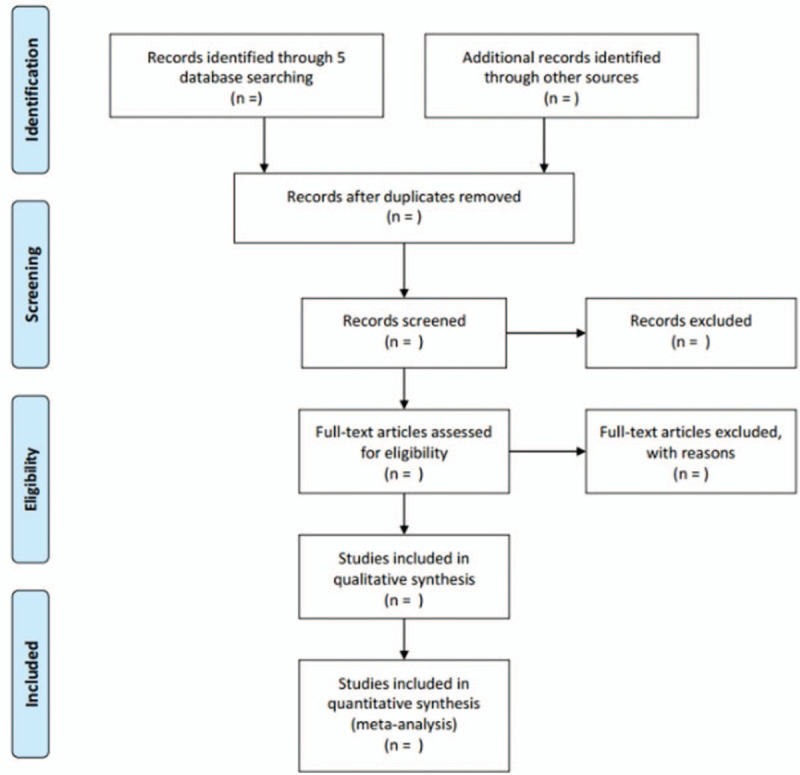
Flow diagram of the study selection process.

The titles and abstracts of all searched studies will be identified by 2 independent reviewers (HT and YH) according to the inclusion criteria. The full text will be reviewed if necessary. Any disagreements will be resolved through discussion with a third reviewer. Excluded studies will be listed in a table with reasons for their exclusion. The study selection procedure is shown in Figure 1.

#### 2.3.2 Data extraction and management

A standard data extraction form will be created before data extraction. Data extraction will be conducted independently by 2 reviewers using a predesigned, pilot-tested extraction form, and dissenting opinions will be settled by a third party. For each study, the following baseline demographic and clinical characteristics were extracted from each study: the first author's name, year of publication, study design, trial phase, underlying malignancy, population size, treatment, and dosing regimens, median age, median treatment duration, number of patients available for analysis, outcomes and number of relevant AEs. The latest report will be included when a same trial was described by multiple publications. If the data of selected studies will be not available in the publications, we will contact the corresponding authors for more information.

#### 2.3.3 Assessment of risk of bias

Two review authors will independently assess the risk of bias for the included studies using the Cochrane Handbook for Systematic Reviews of Interventions (RevMan, version 5.3; Cochrane Collaboration, 2011) to assess the quality and risk of bias for each selected article. Two authors reviewed all selected articles and reported their evaluations as “high”, “low”, or “unclear” for each option, based on the following standards:

(1) selection bias (ie, whether there was adequate generation of the randomization sequence and whether the allocation concealment was satisfactory);
(2) blinding (ie, performance bias and detection bias such as whether there was blinding of the participants, personnel, and outcome assessments);
(3) attrition bias (ie, whether incomplete outcome data were sufficiently assessed and addressed);
(4) reporting bias (ie, whether there was evidence of selective outcome reporting); and
(5) other biases (ie, whether the study was free of other problems that could increase the risk of bias).

To improve accuracy, any disagreements were resolved by a third-party author's opinion. Disagreements between the 2 review authors over the risk of bias in particular studies will be resolved by a third reviewer's opinion.

#### 2.3.4 Measures of treatment effect

For dichotomous data, risk ratio with 95% confidence intervals (CIs) will be used for analysis. For continuous data, mean difference with 95% CIs will be used for analysis. Standardized mean difference with 95% CIs will be used if different scales were used to measure a certain outcome variable.

#### 2.3.5 Strategy for data synthesis

After the assessment of included studies’ bias, then data will be collected and a meta-analysis will be conducted. Dichotomous data were pooled as relative risk in a random-effects model. Time-to-event data were pooled as the hazard ratio using the generic inverse-variance method. In the case of missing SD of the mean change from baseline, it was calculated from the SE or the 95% confidence interval (CI) according to Altman and Bland. We will assess the presence of publication bias using funnel plots inspection and Egger test. All analyses were performed in Review Manager version 5.3 and Excel 2010. Heterogeneity was assessed using the *χ*2-test, where *P* values less than .1 were considered statistically significant, and its extent was measured using the *I*^2^-test.

#### 2.3.6 Analysis of subgroups or subsets

Subgroup analysis will be performed to interpret the heterogeneity if it is possible. In our study, we will pool the total effect for all types of cancers as well as the different effect in each type of cancer used the prescription of ipilimumab combined with nivolumab. Meanwhile, if the necessary data are available, subgroup analyses will also be done for people with different line chemotherapy, age (<30, 30–65, >65 years).

#### 2.3.7 Sensitivity analysis and summary of evidence

If it is possible, the sensitivity analysis will be conducted to verify the robustness of the primary efficacy and safety. We will also evaluate the impacts of methodological quality, sample size and missing data. We will assess the quality of evidence for all outcomes by the Grading of Recommendations Assessment Development and Evaluation criteria.^[18]^

#### 2.3.8 Ethics and dissemination

Because data will not be collected from individual patients directly and privacy will also not be involved, ethics approval will not be required. The results will be disseminated through publication in a peer-reviewed scientific journal. The essential protocol amendments will be documented in the full review.

#### 2.3.9 Patient and public involvement

In our study, data will not be collected from patients directly but from published studies which will be selected and screen in main databases.

## 3 Conclusion

The combination of ipilimumab and nivolumab has shown the effectiveness in various cancer as a new treatment strategy. In this systematic review, we will provide an assessment of the current state of the combination of ipilimumab and nivolumab for cancer patients. The process of conducting this review will be divided into 4 parts: identification, study inclusion, data extraction, and data synthesis. The conclusions of this review may provide comparative efficacy and safety data to aid decision making by clinicians, patients who suffered from the cancer, and policymakers.

### 3.1 Review status

Preliminary searches.

## Author contributions

Hongxuan Tong and Tao Lu designed the systematic review. Hongxuan Tong drafted the protocol and Yu Hao revised the manuscript. Yu Hao and Kaili Wang will independently screen the potential studies, extract data, assess the risk of bias and finish data synthesis. Lekang Xiang will arbitrate any disagreements during the review. All authors approved the publication of the protocol.

Hongxuan Tong orcid: 0000-0003-4788-1768.
